# Supplementary material for: Climatic niche shift and possible future spread of the invasive South African Orchid Disa bracteata in Australia and adjacent areas
Source: PeerJ. 2018 Dec 20;6:e6107. doi: 10.7717/peerj.6107 (PMC6304271; doi:10.7717/peerj.6107)
Supplement: File S2 — Variables used for modeling are marked with bold. All variables were used for PCA. Topographic variables marked with “Calculated*” as a source were calculated with “raster” package in R using function “terrain” on altitude dataset. [file peerj-06-6107-s002.docx]

**Appendix 2**

**Title**: Climatic niche shift and possible future spread of the invasive South African Orchid *Disa bracteata* in Australia and adjacent areas

**Authors**: Kamil Konowalik^1*^ & Marta Kolanowska^2,3^

^1^Department of Plant Biology, Institute of Biology, Wrocław University of Environmental and Life Sciences, Kożuchowska 5b, 51-631 Wrocław, Poland

^2^Department of Plant Taxonomy & Nature Conservation, Faculty of Biology, The University of Gdansk, Wita Stwosza 59, PL-80-308 Gdansk, Poland

^3^Department of Biodiversity Research, Global Change Research Institute AS CR, Bělidla 4a, 603 00 Brno, Czech Republic

^*^author for correspondence: e-mail: kamil.konowalik@upwr.edu.pl

Variables used by MaxentVariableSelection to choose appropriate set of variables and betamultiplier. Topographic variables marked with “Calculated*” as a source were calculated with “raster” package in R using function “terrain” on altitude dataset. Variables used for modeling are marked with bold. All variables were used for PCA.

| Variable | Full name | Source |
| --- | --- | --- |
| Bio 1 | Annual Mean Temperature | Karger et al., 2016 |
| Bio 2 | Mean Diurnal Range (Mean of monthly (max temp - min temp)) | Karger et al., 2016 |
| Bio 3 | Isothermality | Karger et al., 2016 |
| Bio 4 | Temperature Seasonality | Karger et al., 2016 |
| Bio 5 | Maximum Temperature of Warmest Month | Karger et al., 2016 |
| Bio 6 | Minimum Temperature of Coldest Month | Karger et al., 2016 |
| **Bio 7** | **Temperature Annual Range (BIO5-BIO6)** | **Karger et al., 2016** |
| **Bio 8** | **Mean Temperature of Wettest Quarter** | **Karger et al., 2016** |
| Bio 9 | Mean Temperature of Driest Quarter | Karger et al., 2016 |
| Bio 10 | Mean Temperature of Warmest Quarter | Karger et al., 2016 |
| Bio 11 | Mean Temperature of Coldest Quarter | Karger et al., 2016 |
| Bio 12 | Annual Precipitation | Karger et al., 2016 |
| Bio 13 | Precipitation of Wettest Month | Karger et al., 2016 |
| Bio 14 | Precipitation of Driest Month | Karger et al., 2016 |
| **Bio 15** | **Precipitation Seasonality** | **Karger et al., 2016** |
| Bio 16 | Precipitation of Wettest Quarter | Karger et al., 2016 |
| Bio 17 | Precipitation of Driest Quarter | Karger et al., 2016 |
| **Bio 18** | **Precipitation of Warmest Quarter** | **Karger et al., 2016** |
| **Bio 19** | **Precipitation of Coldest Quarter** | **Karger et al., 2016** |
| BDTICM_M_250m_ll | Absolute depth to bedrock (in cm) | Hengl et al., 2014 |
| OCSTHA_M_sd1_250m_ll | Soil organic carbon stock in tonnes per ha 5-15 cm | Hengl et al., 2014 |
| CLYPPT_M_sl1_250m_ll | Clay content (0–2 micro meter) mass fraction in % - 0 cm | Hengl et al., 2014 |
| CLYPPT_M_sl2_250m_ll | Clay content (0–2 micro meter) mass fraction in % - 5 cm | Hengl et al., 2014 |
| CRFVOL_M_sl1_250m_ll | Coarse fragments volumetric in % - 0 cm | Hengl et al., 2014 |
| CRFVOL_M_sl2_250m_ll | Coarse fragments volumetric in % - 5 cm | Hengl et al., 2014 |
| SLTPPT_M_sl1_250m_ll | Silt content (2–50 micro meter) mass fraction in % - 0 cm | Hengl et al., 2014 |
| SLTPPT_M_sl2_250m_ll | Silt content (2–50 micro meter) mass fraction in % - 5 cm | Hengl et al., 2014 |
| **SNDPPT_M_sl1_250m_ll** | **Sand content (50–2000 micro meter) mass fraction in % - 0 cm** | **Hengl et al., 2014** |
| SNDPPT_M_sl2_250m_ll | Sand content (50–2000 micro meter) mass fraction in % - 5 cm | Hengl et al., 2014 |
| CECSOL_M_sl1_250m_ll | Cation exchange capacity of soil in cmolc/kg - 0 cm | Hengl et al., 2014 |
| CECSOL_M_sl2_250m_ll | Cation exchange capacity of soil in cmolc/kg - 5 cm | Hengl et al., 2014 |
| **ORCDRC_M_sl1_250m_ll** | **Soil organic carbon content (fine earth fraction) in g per kg - 0 cm** | **Hengl et al., 2014** |
| ORCDRC_M_sl2_250m_ll | Soil organic carbon content (fine earth fraction) in g per kg - 5 cm | Hengl et al., 2014 |
| **PHIHOX_M_sl1_250m_ll** | **Soil pH x 10 in H2O - 0 cm** | **Hengl et al., 2014** |
| PHIHOX_M_sl2_250m_ll | Soil pH x 10 in H2O - 5 cm | Hengl et al., 2014 |
| PHIKCL_M_sl1_250m_ll | Soil pH x 10 in KCl - 0 cm | Hengl et al., 2014 |
| PHIKCL_M_sl2_250m_ll | Soil pH x 10 in KCl - 5 cm | Hengl et al., 2014 |
| Aspect | Aspect | calculated* |
| Flowdir | Flow direction | calculated* |
| Roughness | Roughness | calculated* |
| Slope | Slope | calculated* |
| TPI | Topographic Position Index | calculated* |
| TRI | Terrain Ruggedness  Index | calculated* |
| Altitude | Altitude | Hijmans et al., 2005 |
| Potveg | Potential vegetation | Ramankutty & Foley 1999 |
| sq1 | Nutrient availability | Fischer et al. 2008 |
| sq2 | Nutrient retention capacity | Fischer et al. 2008 |
| sq4 | Oxygen availability to roots | Fischer et al. 2008 |
| sq5 | Excess salts | Fischer et al. 2008 |

Models for year 2070 that were chosen to compute ensemble model for 2070. Each model covers all four representative concentration pathways - RCPs (rcp26, rcp45, rcp60, rcp85), and each bioclimatic variable of particular RCP was averaged separately.

| No. | Name of the model |
| --- | --- |
| 1 | BCC-CSM1-1 |
| 2 | CCSM4 |
| 3 | GISS-E2-R |
| 4 | HadGEM2-AO |
| 5 | HadGEM2-ES |
| 6 | IPSL-CM5A-LR |
| 7 | MIROC-ESM-CHEM |
| 8 | MIROC-ESM |
| 9 | MIROC5 |
| 10 | MRI-CGCM3 |
| 11 | NorESM1-M |
